# Novel blood-based *FUT7* DNA methylation is associated with lung cancer: especially for lung squamous cell carcinoma

**DOI:** 10.1186/s13148-022-01389-2

**Published:** 2022-12-03

**Authors:** Yifei Fang, Yunhui Qu, Longtao Ji, Hao Sun, Jiaqi Li, Yutong Zhao, Feifei Liang, Zhi Wang, Jiao Su, Jingjing Liu, Liping Dai, Songyun Ouyang

**Affiliations:** 1 Department of Respiratory and Sleep Medicine, The First Affiliated Hospital of Zhengzhou University, Zhengzhou, 450052 Henan China; 2 Department of Clinical Laboratory, The First Affiliated Hospital of Zhengzhou University and Key Clinical Laboratory of Henan Province, Zhengzhou, 450052 Henan China; 3 Henan Institute of Medical and Pharmaceutical Sciences, Zhengzhou University, Zhengzhou, 450052 Henan China; 4 BGI College, Zhengzhou University, Zhengzhou, 450052 Henan China; 5 Henan Key Medical Laboratory of Tumor Molecular Biomarkers, Zhengzhou University, Zhengzhou, 450052 Henan China; 6 Department of Radiotherapy, The First Affiliated Hospital of Zhengzhou University, Zhengzhou, 450052 Henan China

**Keywords:** FUT7, Lung cancer, DNA methylation, Biomarker, Diagnosis

## Abstract

**Background:**

The death rate of lung cancer (LC) ranks first in the world. Changes of DNA methylation in peripheral blood may be related to malignant tumors. It is necessary to explore blood-based biomarkers of methylation to detect LC.

**Methods:**

Mass spectrometry assays were conducted to measure DNA methylation levels of seven CpG sites within *FUT7* gene in the peripheral blood of 428 patients with LC, 233 patients with benign pulmonary nodule (BPN) and 862 normal controls (NC). The odds ratios (ORs) of all CpG sites were evaluated for their risk to LC using per SD change and tertiles analyses by logistic regression. The predictive ability of the seven *FUT7* CpG sites and risk factors were evaluated by receiver operating characteristic curve (ROC).

**Results:**

The methylation levels of seven CpG sites of *FUT7* in LC were significantly lower than that in NC (*P* < 0.05). The per SD decrement of methylation level in CpG_1-7 was significantly associated with 65%, 38%, 59%, 46%, 23%, 20% and 68% higher risk for LC versus NC, respectively, and the adjusted ORs (95% CI) were 2.92 (2.17–3.96), 1.76 (1.29–2.38), 2.83 (2.09–3.82), 3.00 (2.17–4.16), 1.81 (1.35–2.43), 1.48 (1.11–1.97) and 3.04 (2.23–4.16) for the lowest tertiles of methylation level in CpG_1-7 compared with the top tertiles, respectively. The area under the curve (AUC) of FUT7_CpG_1-7 was 0.659 (CI 0.626–0.693), 0.792 (CI 0.736–0.848) and 0.729 (CI 0.665–0.792) in distinguishing LC versus NC, LUSC versus NC and LUSC versus BPN.

**Conclusions:**

Our study revealed an association between *FUT7* hypomethylation and LC, especially for LUSC, which provides novel support for the blood-based methylation signatures as potential marker for assessing lung cancer risk.

**Supplementary Information:**

The online version contains supplementary material available at 10.1186/s13148-022-01389-2.

## Background

Lung cancer (LC) is the leading cause of death from cancer, with 1.8 million deaths worldwide in 2020 [1]. The mortality rate of LC in China is among the highest globally, and it remains on the rise [2]. The 5-year relative survival rate for LC is only 6%, mainly due to diagnosis at late stages of 57% patients, and the 5-year survival rate for localized stage disease is 59% [1]. Therefore, initial diagnosis and proper treatment are efficient way to improve the survival of LC patients. Low-dose computed tomography (LDCT) screening has been proven to reduce LC mortality by 20% in high-risk populations, but the high false positives rate and overdiagnosis should also be concerned [3, 4]. In addition, the sensitivity of LDCT may be severely affected by tumor size and location, varying in a wide range (60–80%) [5, 6].

Epigenetics plays a vital role in the occurrence and development of many diseases, and its characteristic is to regulate gene expression without changing the DNA sequence. DNA methylation performs a vital epigenetic mechanism that involves the regulation of X chromosome inactivation, genomic imprinting, tissue-specific gene expression and a variety of disorders [7, 8].

Genome-wide hypomethylation and hypermethylation changes were found in LC, which may be used as markers [9]. For example, hypermethylation of SHOX2 and p16/CDKN2A was reported for early detection of LC [10–12]. Fucosyltransferases (FUTs), catalyzing the transfer of GDP-fucose residues to the receptor molecules to complete fucosylation, participate in various biological processes, including tumor progression, cell adhesion and differentiation [13–15]. As far as we know, there are 13 genes in FUT family, divided into four subfamilies based on glycosidic bonds, namely α1,2-, α1,3/4-, α1,6- and protein O-fucosylation [16]. *FUT7* belongs to the α1,3/4-fucosyltransferase family and catalyzes the synthesis of α1,3-fucose [17]. Evidence is mounting that the expression of *FUT7* is increased in liver cancer, lung cancer, breast cancer and other solid tumors [18, 19]. According to previous studies, *FUT7* may promote the process of cancers via EGFR/AKT/mTOR signaling pathway and MAPK and PI3K/Akt signaling pathway [19, 20]. However, there is rare report about the association between blood-based *FUT7* methylation and lung cancers. The purpose of our study is to explore the relationship of lung cancer with *FUT7* methylation in peripheral blood and the detection value of *FUT7* methylation in LC patients.

## Material and methods

### Study population

A total of 1523 patients were included in the study, of which 428 patients with LC, 233 patients with BPN, and 862 unrelated self-reported healthy individuals were consecutively recruited from the First Affiliated Hospital of Zhengzhou University between January 2018 and January 2021, and approved by the Ethics Committee of the First Affiliated Hospital of Zhengzhou University (2021-KY-1057-002). The diagnosis of LC and BPN was confirmed by thoracic surgery or pneumocentesis followed by histopathological diagnosis, and the blood samples of these patients were collected before surgery and any cancer-related treatments. The detailed characteristics of participants are shown in Table 1.

**Table 1:** Characteristics of study participants

Variables	LC (*n* = 428)	BPN (*n* = 233)	NC (*n* = 862)
Age, year	60.13 ± 10.48	53.59 ± 12.50	57.27 ± 12.98
Male, *n* (%)	241 (56.31%)	148 (63.52%)	585 (67.87%)
History of chronic lung diseases, *n* (%)	34 (7.94%)	31 (13.31%)	–
Personal tumor history, *n* (%)	15 (3.51%)	9 (3.86%)	–
Family tumor history, *n* (%)	64 (14.95%)	27 (11.59%)	–
Smoking, *n* (%)	150 (35.05%)	67 (28.76%)	–
Alcohol drinking, *n* (%)	95 (22.20%)	49 (21.03%)	–
Nodule length, mm	31.18 ± 21.29	23.93 ± 17.25	–
Tumor types			
Squamous carcinoma, *n* (%)	81 (18.97%)	–	–
Adenocarcinoma, *n* (%)	291 (68.15%)	–	–
Other NSCLC, *n* (%)	26 (6.08%)	–	–
Small cell carcinoma, *n* (%)	29 (6.79%)	–	–
Clinical stage			
I, *n* (%)	154 (37.75%)	–	–
II, *n* (%)	18 (4.41%)	–	–
III, *n* (%)	103 (25.25%)	–	–
IV, *n* (%)	133 (32.60%)	–	–
Unstaged	20 (4.67%)	–	–
BPN types			
Tuberculosis, *n* (%)	–	23 (9.87%)	–
Mycotic infection, *n* (%)	–	13 (5.58%)	–
Chronic inflammation, *n* (%)	–	100 (42.92%)	–
Pulmonary fibrosis, *n* (%)	–	2 (0.86%)	–
Inflammatory pseudotumor, *n* (%)	–	5 (2.15%)	–
Hamartoma, *n* (%)	–	11 (4.72%)	–
Sclerotic pulmonary cytomas, *n* (%)	–	15 (6.44%)	–
Granuloma, *n* (%)	–	6 (2.58%)	–
Others/unknown, *n* (%)	–	11 (4.72%)	–

*LC* lung cancer, *BPN* benign pulmonary nodule, *NC* normal control, *NSCLC* non-small cell lung cancer

### Sample processing

All the peripheral blood samples were collected by EDTA blood collection tubes and kept at 4 °C for less than 24 h before the storage at − 80 °C for future usage. DNA Extraction Kit (TANTICA, Nanjing, China) was used to extract DNA from whole blood and further bisulfite-converted utilized EZ-96 DNA Methylation Gold Kit according to standard protocol (Zymo Research, Orange, U.S.). All the samples were processed in parallel.

### MALDI-TOF mass spectrometry

Bisulfite converted DNA of all participants was amplified by bisulfite-specific primers. The sequence of target region is shown in Additional file 1: S1. There is no single nucleotide polymorphism (SNP) nor CpG site in the primers. Forward primer: 5′-aggaagagagTAAAATGTTGGGATTATAGTTTGGG-3′, reverse primer: 5′-cagtaatacgactcactatagggagaaggctAAAACCAAATTCCTTCTTCTACACC-3′. Upper case letters presented the sequence specific regions, and the unspecific tags were shown in lower case letters. The PCR products were analyzed by a matrix-assisted laser desorption ionization time-of-flight (MALDI-TOF) mass spectrometry for the semi-quantitative measurements of the DNA methylation intensity at the single CpG resolution (Agena Bioscience, California, U.S.). Briefly, the PCR amplified products were incubated with Shrimp Alkaline Phosphatase (SAP) and further transcribed to RNA by T7 transcriptase according to the standard protocol of Agena EpiTyper assay (Agena Bioscience, California, U.S.). The RNA was digested by RNase into small fragments and then cleaned the ions by resin. The final products were dispensed on a 384 SpectroCHIP. The DNA methylation levels were semi-quantitatively determined by comparing the intensities of methylated and non-methylated segments. The data were collected by SpectroACQUIRE v3.3.1.3 software and visualized by EpiTyper v1.3 software.

### Statistical analysis

All statistical data were analyzed by SPSS Statistics 23.0 software and GraphPad Prism 9.0. The mean ± SD or the median (25th percentile, 75th percentile) and the number (%) were utilized to describe continuous data and categorical data, respectively. Mann–Whitney *U* test was adopted to compare the methylation level in different clinical categories due to the non-normal distribution of the data and the Z value was calculated to make the results adequate and convincing. Chi-squared test was applied for categorical data. By presenting the 95% confidence interval (CI) and the odds ratios (ORs) with adjustment for covariates (age, sex, smoking, alcohol drinking, history of chronic lung diseases and personal tumor history), the association between *FUT7* methylation and LC were assessed via logistic regression analyses. In the assessing of *FUT7* methylation, we established models for both continuous data per SD decrement and categorical data by utilizing tertiles with the highest tertile (T3) as the reference group. The tests for linear trend were performed by entering the tertiles of each category of seven *FUT7* CpG sites as continuous data in the models. The predictive ability of seven *FUT7* CpG sites (FUT7_CpG_1-7) was evaluated via the corresponding area under curve (AUC) of receiver operating characteristic curve (ROC) with 95% CI. A two-sided *P* < 0.05 was statistically significant.

## Results

### The methylation levels of *FUT7* in LC were lower than that in NC and BPN

**Fig. 1:**
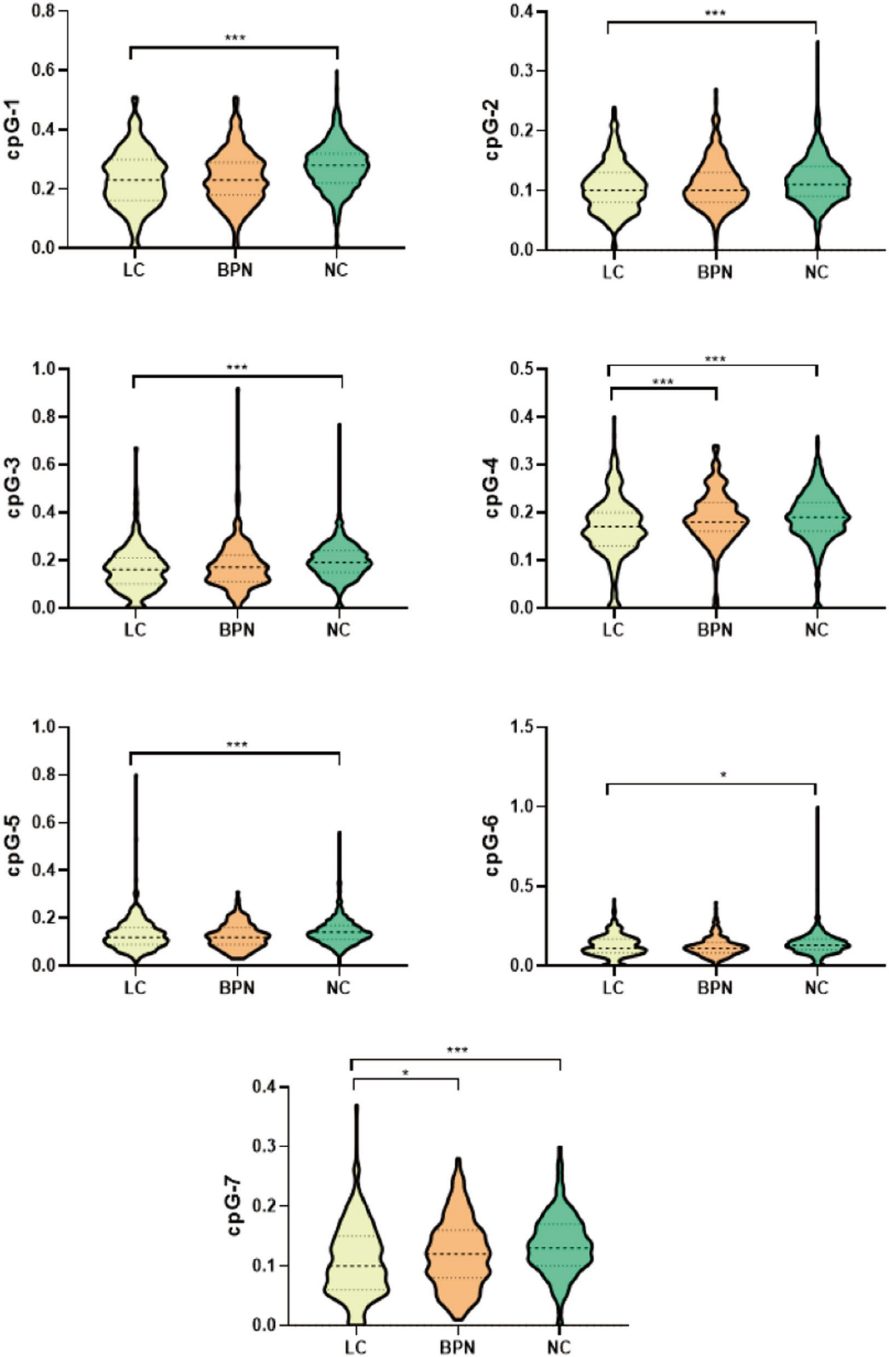
The methylation levels of FUT7 in LC, BPN and NC. *LC* lung cancer, *BPN* benign pulmonary nodule, *NC* normal control. *P < 0.05; ***P < 0.001. P: Mann–Whitney-*U* test was used to continuous data

As shown in Table 2 and Fig. 1, the methylation levels of seven sites of *FUT7* in LC were significantly lower than that in NC (*P* < 0.05). The methylation levels of CpG-4 and CpG-7 were lower in LC than in BPN (*P* < 0.05), and no significant differences in other sites (Table 2; Fig. 1).

**Table 2:** The methylation levels of FUT7_CpG_1-7 in LC, BPN and NC

Variables	LC (*n* = 428)	BPN (*n* = 233)	NC (*n* = 862)	*Z*_1_ value	*P*_1_ value	*Z*_2_ value	*P*_2_ value
FUT7_CpG_1	0.230 (0.168–0.300)	0.230 (0.180–0.290)	0.280 (0.220–0.320)	7.986	< 0.001***	0.458	0.647
FUT7_CpG_2	0.100 (0.080–0.130)	0.100 (0.080–0.130)	0.110 (0.090–0.140)	5.244	< 0.001***	1.926	0.055
FUT7_CpG_3	0.160 (0.100–0.210)	0.170 (0.110–0.220)	0.190 (0.150–0.240)	6.753	< 0.001***	1.587	0.113
FUT7_CpG_4	0.170 (0.130–0.200)	0.180 (0.160–0.220)	0.190 (0.160–0.220)	6.359	< 0.001***	4.035	< 0.001***
FUT7_CpG_5	0.120 (0.090–0.160)	0.120 (0.090–0.160)	0.140 (0.110–0.170)	3.227	0.001**	1.335	0.182
FUT7_CpG_6	0.110 (0.080–0.170)	0.110 (0.080–0.140)	0.130 (0.100–0.170)	2.570	0.010*	0.767	0.443
FUT7_CpG_7	0.100 (0.060–0.150)	0.120 (0.080–0.160)	0.130 (0.100–0.170)	8.167	< 0.001***	2.387	0.017*

*LC* lung cancer, *BPN* benign pulmonary nodule, *NC* normal control

*Z*_1_, *P*_1_: comparison between LC and NC using Mann–Whitney-*U* test; *Z*_2_, *P*_2_: comparison between LC and BPN using Mann–Whitney-*U* test

*P < 0.05; **P < 0.01; *** *P* < 0.001

### Association between *FUT7* methylation and LC patients

**Fig. 2:**
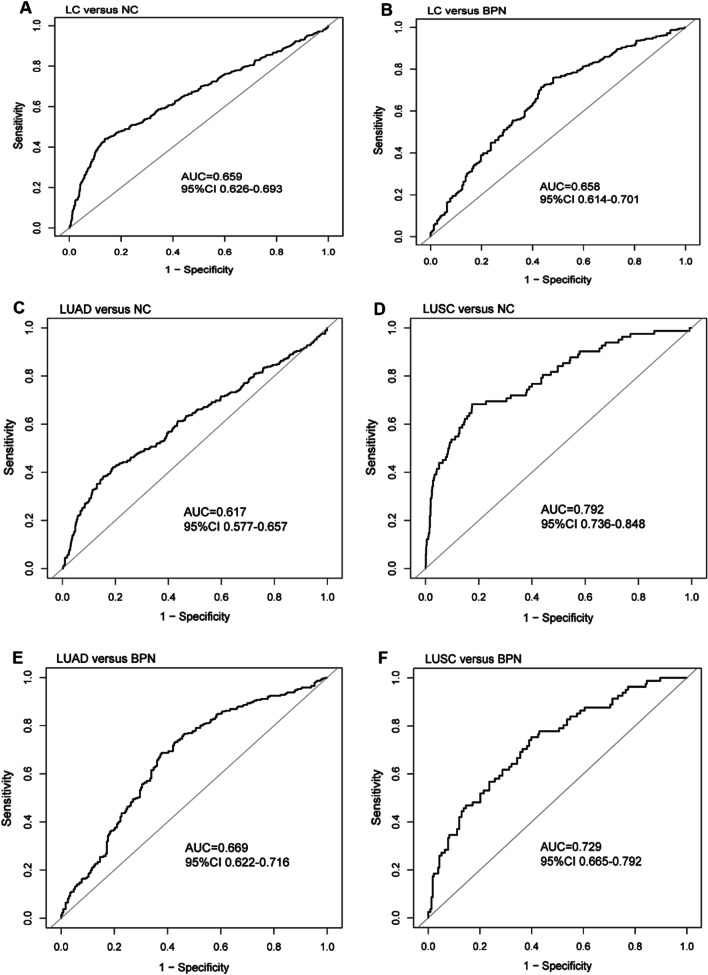
The diagnostic capability of FUT7_CpG_1-7. **A** ROC for LC versus NC. **B** ROC for LC versus BPN. **C** ROC for LUAD versus NC. **D** ROC for LUSC versus NC. **E** ROC for LUAD versus BPN. **F** ROC for LUSC versus BPN. *ROC* receiver operating characteristic curve, *AUC* area under the curve, *NC* normal control, *BPN* benign pulmonary nodule, *LC* lung cancer, *LUAD* lung adenocarcinoma, *LUSC* lung squamous cell carcinoma

The per SD decrement and tertiles were conducted to evaluate the ORs of seven CpG sites in *FUT7* for the risk of LC by multivariate logistic regression. In adjusted model (adjusted age and sex), the per SD decrement of methylation level in CpG_1-7 was significantly associated with 65%, 38%, 59%, 46%, 23%, 20% and 68% higher risk for LC versus NC, respectively, and the adjusted OR (95% CI) was 2.92 (2.17–3.96), 1.76 (1.29–2.38), 2.83 (2.09–3.82), 3.00 (2.17–4.16), 1.81 (1.35–2.43), 1.48 (1.11–1.97) and 3.04 (2.23–4.16) for the lowest tertile of methylation level in CpG_1-7 compared with the top tertile, respectively (*P* for trend < 0.05) (Table 3). After adjustment for age, gender smoking, alcohol drinking, history of chronic lung diseases and personal tumor history in adjusted model, the per SD decrement of methylation level in CpG_4 was significantly associated with 32% higher risk for LC versus BPN (Table 3). To explore the value of *FUT7* methylation in the detection of LC, the combination analyses of seven CpG sites were performed. ROC analysis showed AUC of 0.659 (95% CI 0.626–0.693) and 0.658 (CI 0.614–0.701) in distinguishing LC from NC (Fig. 2A) and LC versus BPN (Fig. 2B).

**Table 3:** Odds ratio of LC versus NC or BPN according to continuous or tertiles of FUT7_CpG_1-7

Variables	LC versus NC					LC versus BPN			
	Crude		Adjusted model^a^			Crude		Adjusted model^b^	
	OR (95% CI)	*P* value	OR (95% CI)	*P* value		OR (95% CI)	*P* value	OR (95% CI)	*P* value
FUT7_CpG_1 (Per 1 SD decrease)	1.62 (1.43–1.84)	< 0.001***	1.65 (1.45–1.88)	< 0.001***		1.04 (0.89–1.22)	0.646	1.04 (0.87–1.23)	0.699
Tertiles of FUT7_CpG_1									
T3 (≥ 0.300)	1.00 (reference)		1.00 (reference)		T3 (≥ 0.280)	1.00 (reference)		1.00 (reference)	
T2 (0.230–0.290)	1.14 (0.83–1.57)	0.405	1.21 (0.88–1.67)	0.237	T2 (0.190–0.270)	1.02 (0.69–1.52)	0.924	1.08 (0.70–1.66)	0.719
T1 (< 0.230)	2.77 (2.07–3.71)	< 0.001***	2.92 (2.17–3.96)	< 0.001***	T1 (< 0.190)	1.12 (0.76–1.67)	0.565	1.12 (0.73–1.73)	0.608
*P* for trend		< 0.001***		< 0.001***			0.556		0.611
FUT7_CpG_2 (Per 1 SD decrease)	1.39 (1.23–1.57)	< 0.001***	1.38 (1.21–1.57)	< 0.001***		1.17 (0.99–1.37)	0.055	1.15 (0.97–1.37)	0.115
Tertiles of FUT7_CpG_2									
T3 (≥ 0.130)	1.00 (reference)		1.00 (reference)		T3 (≥ 0.120)	1.00 (reference)		1.00 (reference)	
T2 (0.100–0.120)	1.12 (0.81–1.55)	0.496	1.08 (0.77–1.50)	0.668	T2 (0.090–0.110)	1.11 (0.73–1.67)	0.630	1.10 (0.70–1.72)	0.689
T1 (< 0.100)	1.83 (1.36–2.47)	< 0.001***	1.76 (1.29–2.38)	< 0.001***	T1 (< 0.090)	1.27 (0.86–1.86)	0.233	1.22 (0.80–1.87)	0.358
*P* for trend		< 0.001***		< 0.001***			0.229		0.354
FUT7_CpG_3 (Per 1 SD decrease)	1.56 (1.37–1.79)	< 0.001***	1.59 (1.39–1.83)	< 0.001***		1.14 (0.97, 1.33)	0.115	1.130 (0.95, 1.35)	0.172
Tertiles of FUT7_CpG_3									
T3 (≥ 0.210)	1.00 (reference)		1.00 (reference)		T3 (≥ 0.190)	1.00 (reference)		1.00 (reference)	
T2 (0.150–0.200)	1.37 (1.01–1.86)	0.045*	1.34 (0.98–1.82)	0.068	T2 (0.120–0.180)	0.99 (0.67–1.46)	0.941	0.96 (0.62–1.47)	0.836
T1 (< 0.150)	2.71 (2.02–3.62)	< 0.001***	2.83 (2.09–3.82)	< 0.001***	T1 (< 0.120)	1.24 (0.84–1.83)	0.279	1.17 (0.76–1.79)	0.477
*P* for trend		< 0.001***		< 0.001***			0.286		0.489
FUT7_CpG_4 (Per 1 SD decrease)	1.46 (1.29–1.64)	< 0.001***	1.46 (1.29–1.66)	< 0.001***		1.40 (1.19–1.66)	< 0.001***	1.32 (1.10–1.58)	0.003**
Tertiles of FUT7_CpG_4									
T3 (≥ 0.210)	1.00 (reference)		1.00 (reference)		T3 (≥ 0.200)	1.00 (reference)		1.00 (reference)	
T2 (0.160–0.200)	1.35 (0.98–1.86)	0.066	1.32 (0.96–1.83)	0.093	T2 (0.150–0.190)	1.19 (0.81–1.75)	0.381	1.00 (0.66–1.53)	0.991
T1 (< 0.160)	2.96 (2.16–4.05)	< 0.001***	3.00 (2.17–4.16)	< 0.001***	T1 (< 0.150)	2.51 (1.64–3.83)	< 0.001***	2.15 (1.35–3.43)	0.001**
*P* for trend		< 0.001***		< 0.001***			< 0.001***		0.002**
FUT7_CpG_5 (Per 1 SD decrease)	1.24 (1.09–1.41)	0.001**	1.23 (1.08–1.40)	0.002**		0.89 (0.75–1.06)	0.184	0.90 (0.74–1.09)	0.263
Tertiles of FUT7_CpG_5									
T3 (≥ 0.150)	1.00 (reference)		1.00 (reference)		T3 (≥ 0.140)	1.00 (reference)		1.00 (reference)	
T2 (0.110–0.140)	0.85 (0.63–1.15)	0.292	0.84 (0.62–1.15)	0.274	T2 (0.100–0.130)	0.83 (0.55–1.24)	0.364	0.81 (0.52–1.26)	0.353
T1 (< 0.110)	1.82 (1.37–2.41)	< 0.001***	1.81 (1.35–2.43)	< 0.001***	T1 (< 0.100)	0.83 (0.56–1.22)	0.331	0.79 (0.52–1.22)	0.290
*P* for trend		< 0.001***		< 0.001***			0.342		0.298
FUT7_CpG_6 (Per 1 SD decrease)	1.18 (1.04–1.35)	0.011*	1.20 (1.05–1.37)	0.007**		0.94 (0.80–1.10)	0.443	0.968 (0.809–1.158)	0.719
Tertiles of FUT7_CpG_6									
T3 (≥ 0.150)	1.00 (reference)		1.00 (reference)		T3 (≥ 0.140)	1.00 (reference)		1.00 (reference)	
T2 (0.100–0.140)	0.73 (0.54–0.98)	0.037*	0.72 (0.53–0.97)	0.033*	T2 (0.090–0.130)	0.46 (0.31–0.70)	< 0.001***	0.52 (0.34–0.81)	0.004**
T1 (< 0.100)	1.45 (1.09–1.92)	0.010*	1.48 (1.11–1.97)	0.008**	T1 (< 0.090)	0.69 (0.46–1.04)	0.076	0.74 (0.48–1.15)	0.178
*P* for trend		0.011*		0.009**			0.032*		0.097
FUT7_CpG_7 (Per 1 SD decrease)	1.66 (1.46–1.89)	< 0.001***	1.68 (1.47–1.92)	< 0.001***		1.21 (1.03–1.42)	0.018*	1.18 (0.99–1.40)	0.071
Tertiles of FUT7_CpG_7									
T3 (≥ 0.150)	1.00 (reference)		1.00 (reference)		T3 (≥ 0.140)	1.00 (reference)		1.00 (reference)	
T2 (0.100–0.140)	0.96 (0.70–1.31)	0.779	0.95 (0.69–1.31)	0.757	T2 (0.080–0.130)	1.05 (0.71–1.55)	0.818	0.99 (0.64–1.52)	0.956
T1 (< 0.100)	2.93 (2.17–3.96)	< 0.001***	3.04 (2.23–4.16)	< 0.001***	T1 (< 0.080)	1.67 (1.11–2.51)	0.014*	1.50 (0.96–2.35)	0.077
*P* for trend		< 0.001***		< 0.001***			0.018*		0.094

Adjusted model^a^ was adjusted for age, sex

Adjusted model^b^ was adjusted for age, sex, smoking, alcohol drinking, history of chronic lung diseases, personal tumor history

*P < 0.05; **P < 0.01; ***P < 0.001

### The predictive ability of FUT7_CpG_1-7 in LUAD and LUSC patients

**Fig. 3:**
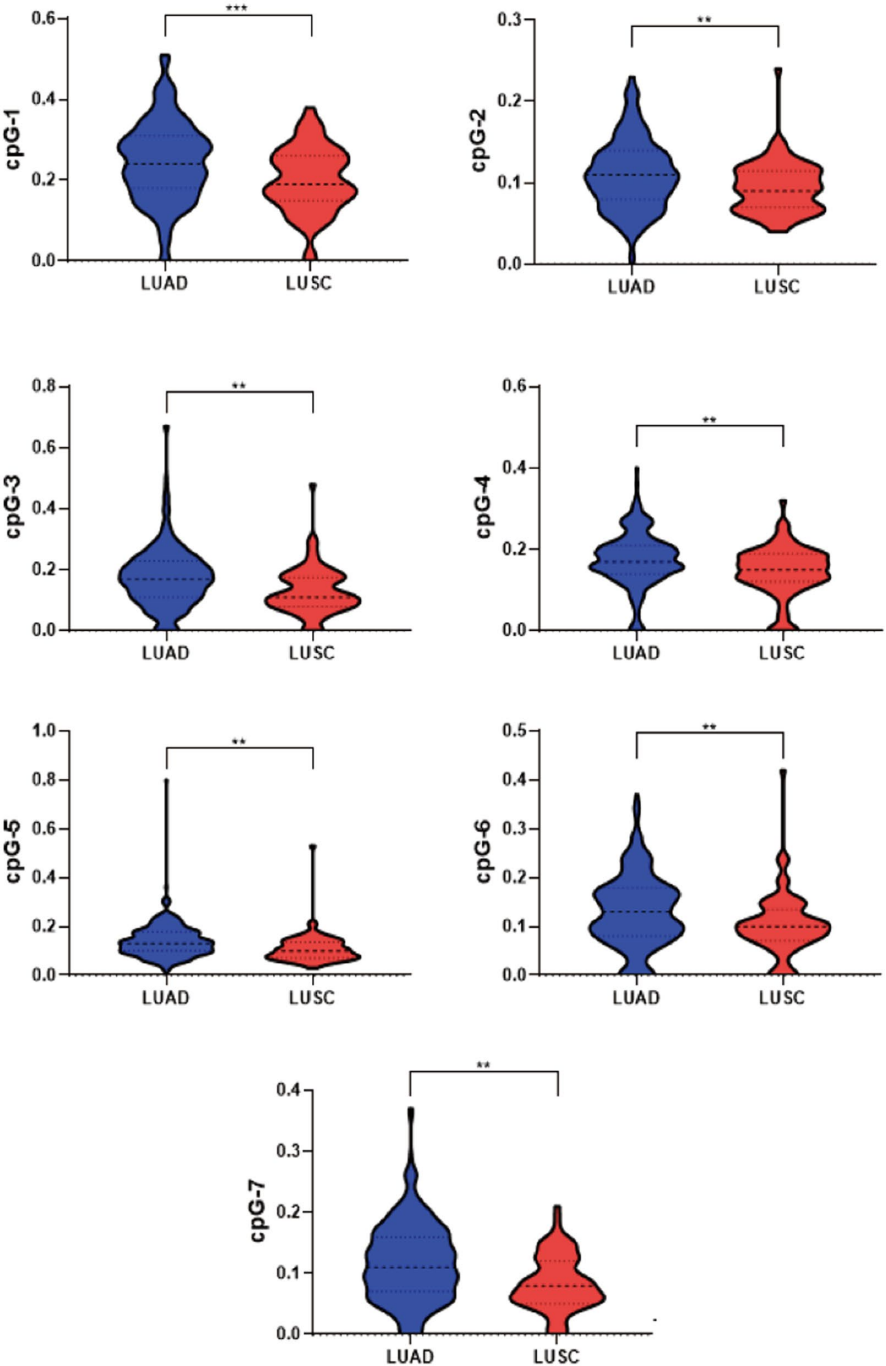
The methylation levels of FUT7_CpG_1-7 in LUAD and LUSC. LUAD lung adenocarcinoma, *LUSC* lung squamous cell carcinoma. **P < 0.01; ***P < 0.001. *P*: Mann–Whitney-*U* test was used to continuous data

There are 291 LUAD and 81 LUSC patients in our study as shown in Table 4. Compared with LUAD patients, LUSC patients were older and more likely to be men. Besides, patients in LUSC had higher rates of smoking, alcohol drinking and history of chronic lung diseases. What’s more, patients with LUSC have longer nodule length and later stages than LUAD. The levels of *FUT7* methylation were higher in LUAD patients than that in LUSC patients (Table 4; Fig. 3).

**Table 4:** Comparison between LUAD and LUSC

Variables	LUAD (*n* = 291)	LUSC (*n* = 81)	*P* value
Age, year	59.01 ± 10.68	62.54 ± 7.65	0.006**
Sex			< 0.001***
Male, *n* (%)	127 (43.64%)	75 (92.59%)	
History of chronic lung diseases, *n* (%)	15 (5.16%)	13 (16.05%)	0.001***
Personal tumor history, *n* (%)	9 (3.16%)	4 (5.13%)	0.407
Family tumor history, *n* (%)	43 (15.09%)	15 (18.52%)	0.456
Smoking	68 (23.86%)	55 (67.90%)	< 0.001***
Alcohol drinking, *n* (%)	47 (16.43%)	33 (41.77%)	< 0.001***
FUT7_CpG_1	0.240 (0.180–0.310)	0.190 (0.150–0.260)	< 0.001***
FUT7_CpG_2	0.110 (0.080–0.135)	0.090 (0.070–0.110)	0.002**
FUT7_CpG_3	0.170 (0.110–0.230)	0.110 (0.080–0.170)	< 0.001***
FUT7_CpG_4	0.170 (0.140–0.210)	0.150 (0.120–0.190)	< 0.001***
FUT7_CpG_5	0.130 (0.100–0.175)	0.100 (0.070–0.130)	< 0.001***
FUT7_CpG_6	0.130 (0.080–0.175)	0.100 (0.070–0.130)	0.002**
FUT7_CpG_7	0.110 (0.070–0.160)	0.080 (0.050–0.120)	< 0.001***
Nodule length, mm	25.657 ± 17.893	44.894 ± 22.703	< 0.001***
Clinical stage			< 0.001***
I, *n* (%)	137 (48.24%)	8 (10.81%)	
II, *n* (%)	13 (4.58%)	5 (6.76%)	
III, *n* (%)	49 (17.25%)	35 (47.30%)	
IV, *n* (%)	85 (29.93%)	26 (35.14%)	

*LUAD* lung adenocarcinoma, *LUSC* lung squamous cell carcinoma

*P*: Mann–Whitney-*U* test was used to continuous data and Chi-squared test was applied to categorical data

*P < 0.05; **P < 0.01; ***P < 0.001

Under the condition that BPN was a control group, multivariate logistic regression analyses of adjusted model (adjusted age, sex, smoking, alcohol drinking, history of chronic lung diseases, personal tumor history) demonstrated that each SD decrement of methylation level in CpG_4 and CpG_5 was significantly associated with 24% and 31% higher risk for LUAD. Compared with the top tertiles, the adjusted OR (95% CI) was 1.62 (1.03, 2.55) and 1.74 (1.08, 2.81) for the lowest tertiles of methylation level in CpG_4 and CpG_5 (*P* for trend < 0.05) (Table 5). Meanwhile, the per SD decrement of methylation level in CpG_1, 2, 3, 4 and 7 was significantly associated with a 57%, 72%, 75%, 115% and 115% higher risk for LUSC, respectively (Table 5). Compared with the top tertiles, the adjusted ORs (95% CI) were 2.66 (1.16, 6.09), 2.97 (1.21, 7.29), 2.90 (1.31, 6.43), 5.19 (2.26, 11.92) and 4.52 (1.93, 10.57) for the lowest tertiles of methylation level in CpG_1, 2, 3, 4 and 7, respectively, after assessing the seven *FUT7* CpG sites as tertiles in model 2 (all *P* for trend < 0.05). ROC curves demonstrated the AUC was 0.617 (CI 0.577–0.657), 0.792 (CI 0.736–0.848), 0.669 (CI 0.622–0.716) and 0.729 (CI 0.665–0.792) for seven *FUT7* CpG sites in the discrimination of LUAD versus NC (Fig. 2C), LUSC versus NC (Fig. 2D), LUAD versus BPN (Fig. 2E) and LUSC from BPN (Fig. 2F). The sensitivity, specificity, positive predictive value (PPV), negative predictive value (NPV), positive likelihood ratio (+ LR), and negative likelihood ratio (-LR) are shown in Table 6.

**Table 5:** Odds ratio of LUAD or LUSC versus BPN according to continuous or tertiles of FUT7_CpG_1-7

Variables	LUAD versus BPN					LUSC versus BPN			
	Crude		Adjusted model^a^			Crude		Adjusted model^b^	
	OR (95% CI)	*P* value	OR (95% CI)	*P* value		OR (95% CI)	*P* value	OR (95% CI)	*P* value
FUT7_CpG_1 (Per 1 SD decrease)	0.92 (0.77–1.09)	0.330	0.93 (0.77, 1.12)	0.448		1.56 (1.19–2.06)	0.001**	1.57 (1.14–2.17)	0.006**
Tertiles of FUT7_CpG_1									
T3 (≥ 0.280)	1.00 (reference)		1.00 (reference)		T3 (≥ 0.270)	1.00 (reference)		1.00 (reference)	
T2 (0.200–0.270)	0.86 (0.56–1.31)	0.474	0.89 (0.56–1.40)	0.606	T2 (0.180–0.260)	1.89 (0.94–3.78)	0.074	2.58 (1.10–6.07)	0.029*
T1 (< 0.200)	0.85 (0.56–1.29)	0.434	0.87 (0.55–1.36)	0.530	T1 (< 0.180)	2.67 (1.35–5.26)	0.005**	2.66 (1.16–6.09)	0.021*
*P* for trend		0.439		0.534			0.005**		0.026*
FUT7_CpG_2 (per 1 SD decrease)	1.05 (0.88–1.24)	0.601	1.03 (0.85–1.24)	0.776		1.69 (1.25–2.29)	< 0.001***	1.72 (1.18–2.52)	0.005**
Tertiles of FUT7_CpG_2									
T3 (≥ 0.120)	1.00 (reference)		1.00 (reference)		T3 (≥ 0.120)	1.00 (reference)		1.00 (reference)	
T2 (0.090–0.110)	0.86 (0.56–1.34)	0.502	0.84 (0.53–1.35)	0.474	T2 (0.090–0.110)	3.31 (1.47–7.49)	0.005**	3.13 (1.21–8.11)	0.019*
T1 (< 0.090)	0.93 (0.61–1.40)	0.726	0.88 (0.56–1.37)	0.556	T1 (< 0.090)	3.92 (1.79–8.57)	< 0.001***	2.97 (1.21–7.29)	0.017*
*P* for trend		0.745		0.570			< 0.001***		0.027*
FUT7_CpG_3 (Per 1 SD decrease)	1.01 (0.85–1.20)	0.921	1.03 (0.86–1.24)	0.743		2.01 (1.41–2.87)	< 0.001***	1.749 (1.166, 2.623)	0.007**
Tertiles of FUT7_CpG_3									
T3 (≥ 0.200)	1.00 (reference)		1.00 (reference)		T3 (≥ 0.180)	1.00 (reference)		1.00 (reference)	
T2 (0.130–0.190)	0.88 (0.58–1.35)	0.566	0.84 (0.53–1.33)	0.449	T2 (0.120–0.170)	1.59 (0.76–3.36)	0.222	1.44 (0.60–3.45)	0.415
T1 (< 0.130)	0.85 (0.55–1.29)	0.435	0.90 (0.57–1.41)	0.634	T1 (< 0.120)	3.68 (1.90–7.10)	< 0.001***	2.90 (1.31–6.43)	0.009**
*P* for trend		0.433		0.629			< 0.001***		0.008**
FUT7_CpG_4 (Per 1 SD decrease)	1.26 (1.05–1.50)	0.011*	1.24 (1.02–1.49)	0.029*		2.21 (1.64–2.98)	< 0.001***	2.15 (1.49–3.09)	< 0.001***
Tertiles of FUT7_CpG_4									
T3 (≥ 0.200)	1.00 (reference)		1.00 (reference)		T3 (≥ 0.190)	1.00 (reference)		1.00 (reference)	
T2 (0.160–0.190)	0.93 (0.60–1.44)	0.738	0.83 (0.52–1.33)	0.444	T2 (0.160–0.180)	2.11 (0.96–4.64)	0.062	2.24 (0.89–5.62)	0.087
T1 (< 0.160)	1.74 (1.14–2.65)	0.010*	1.62 (1.03–2.55)	0.037*	T1 (< 0.160)	5.32 (2.64–10.74)	< 0.001***	5.19 (2.26–11.92)	< 0.001***
*P* for trend		0.014*		0.049*			< 0.001***		< 0.001***
FUT7_CpG_5 (Per 1 SD decrease)	1.32 (1.08–1.60)	0.006**	1.31 (1.06–1.62)	0.014*		1.37 (1.02–1.84)	0.035*	1.173 (0.871, 1.579)	0.295
Tertiles of FUT7_CpG_5									
T3 (≥ 0.150)	1.00 (reference)		1.00 (reference)		T3 (≥ 0.140)	1.00 (reference)		1.00 (reference)	
T2 (0.100–0.140)	1.20 (0.78–1.84)	0.407	1.19 (0.75–1.89)	0.453	T2 (0.090–0.130)	1.48 (0.74–2.98)	0.269	1.55 (0.67–3.57)	0.304
T1 (< 0.100)	1.70 (1.09–2.64)	0.017*	1.74 (1.08, 2.81)	0.022*	T1 (< 0.090)	2.30 (1.16–4.56)	0.017*	1.70 (0.74–3.89)	0.209
*P* for trend		0.016*		0.019*			0.014*		0.229
FUT7_CpG_6 (Per 1 SD decrease)	1.17 (0.98–1.40)	0.076	1.14 (0.94–1.37)	0.187		1.34 (1.01–1.77)	0.046*	1.495 (1.045, 2.139)	0.028*
Tertiles of FUT7_CpG_6									
T3 (≥ 0.150)	1.00 (reference)		1.00 (reference)		T3 (≥ 0.130)	1.00 (reference)		1.00 (reference)	
T2 (0.090–0.140)	0.66 (0.43–1.02)	0.062	0.63 (0.40–1.00)	0.050	T2 (0.090–0.120)	1.01 (0.51–1.99)	0.982	0.78 (0.34–1.81)	0.564
T1 (< 0.090)	1.69 (1.07–2.66)	0.025*	1.51 (0.92–2.45)	0.101	T1 (< 0.090)	1.67 (0.89–3.12)	0.112	2.00 (0.90–4.44)	0.089
*P* for trend		0.005**		0.026*			0.113		0.092
FUT7_CpG_7 (Per 1 SD decrease)	1.05 (0.88–1.24)	0.602	1.05 (0.87–1.26)	0.618		2.12 (1.56–2.89)	< 0.001***	2.147 (1.468, 3.140)	< 0.001***
Tertiles of FUT7_CpG_7									
T3 (≥ 0.140)	1.00 (reference)		1.00 (reference)		T3 (≥ 0.130)	1.00 (reference)		1.00 (reference)	
T2 (0.090–0.130)	0.98 (0.63–1.53)	0.935	0.99 (0.62–1.59)	0.981	T2 (0.080–0.120)	2.11 (0.99–4.51)	0.055	2.35 (0.97–5.72)	0.060
T1 (< 0.090)	1.03 (0.68–1.56)	0.877	1.06 (0.68–1.65)	0.805	T1 (< 0.080)	5.19 (2.55–10.57)	< 0.001***	4.52 (1.93–10.57)	< 0.001***
*P* for trend		0.870		0.799			< 0.001***		< 0.001***

Adjusted model^a^ and Adjusted model^b^ were adjusted for age, sex, smoking, alcohol drinking, history of chronic lung diseases, personal tumor history

*P < 0.05; **P < 0.01; ***P < 0.001

**Table 6:** Diagnostic value of CpG1-7 in distinguishing LC from NC or BPN

	Specificity (%)	Sensitivity (%)	Positive-LR	Negative-LR	PPV (%)	NPV (%)
LC versus NC	86.19	44.16	3.20	0.65	61.36	75.66
LUAD versus NC	80.97	42.12	2.21	0.71	42.86	80.51
LUSC versus NC	82.48	68.29	3.90	0.38	96.47	27.05
LC versus BPN	56.65	71.43	1.65	0.50	75.12	51.97
LUAD versus BPN	62.23	68.73	1.82	0.50	69.44	61.44
LUSC versus BPN	60.09	75.31	1.89	0.41	87.50	39.61

*LC* lung cancer, *BPN* benign pulmonary nodule, *NC* normal control, *LUAD* lung adenocarcinoma, *LUSC* lung squamous cell carcinoma, *Positive-LR* positive likelihood ratio, *Negative-LR* negative likelihood ratio, *PPV* positive predictive value, *NPV* negative predictive value

### The association of methylation levels of *FUT7* with clinical characteristics in LC patients

To understand the methylation patterns in 428 LC patients, the methylation levels of *FUT7* stratified by different clinical characteristics were further analyzed. FUT7_CpG_1-7 were significant different in tumor length. Compared to the patients with smaller nodules (tumor length ≤ 3 cm), the LC patients with more than 3 cm tumor length have significantly lower methylation at FUT7_CpG_1-7 (*P* < 0.05; Table VII). The methylation levels of FUT7_CpG_2,3,5,6,7 groups were lower in terms of clinical stage III and IV, lymph nodes and metastasis than the corresponding control group (*P* < 0.05; Table 7), which indicated that hypomethylation of FUT7_CpG_2,3,5,6,7 might be associated with the progress of LC.

**Table 7:** FUT7_CpG_1-7 methylation levels in LC patients with different clinical characteristics

Variables (n)	FUT7_CpG_1	FUT7_CpG_2	FUT7_CpG_3	FUT7_CpG_4	FUT7_CpG_5	FUT7_CpG_6	FUT7_CpG_7
Clinical stage							
Stage I&II (172)	0.243	0.113	0.183	0.175	0.153	0.142	0.122
Stage III&IV (236)	0.227	0.099	0.148	0.165	0.116	0.113	0.101
* P* value	0.091	0.001**	< 0.001***	0.138	< 0.001***	< 0.001***	< 0.001***
Nodule length							
≤ 3 cm (234)	0.247	0.113	0.178	0.175	0.146	0.136	0.124
> 3 cm (153)	0.219	0.095	0.146	0.158	0.113	0.112	0.090
* P* value	0.005**	< 0.001***	< 0.001***	0.009**	< 0.001***	< 0.001***	< 0.001***
Lymph node metastasis number							
0 (189)	0.236	0.110	0.173	0.168	0.147	0.138	0.117
1–3 (225)	0.233	0.101	0.153	0.169	0.119	0.114	0.103
* P* value	0.733	0.016*	0.025*	0.991	< 0.001***	< 0.001***	0.024*
Distant metastasis							
Yes (136)	0.223	0.097	0.146	0.163	0.114	0.112	0.099
No (271)	0.239	0.109	0.172	0.172	0.141	0.132	0.115
* P* value	0.117	0.003**	0.008**	0.169	< 0.001***	0.003**	0.016*

*P*: Mann–Whitney-*U* test was used to continuous data

*P < 0.05; **P < 0.01; ***P < 0.001

## Discussion

Lung cancer is a high malignant carcinoma leading cause of cancer death worldwide. There is an urgent need to search for the sensitive and specific biomarkers of LC to improve the diagnosis, treatment and prognosis assessment. Our results revealed that the methylation level in all CpG sites in *FUT7* was significantly and negatively associated with LC in the presence of NC as control group. In the case of BPN as control group, the hypomethylation levels of FUT7_CpG_4,5 were associated with increasing risk of LUAD. Furthermore, the increased risk of LUSC was associated with the decreased methylation levels of FUT7_CpG_1,2,3,4,7, and FUT7_CpG_1-7 showed good predictive ability in LUSC. In addition, the hypomethylation of FUT7_CpG_2,3,5,6,7 may associate with the progress of lung cancer.

*FUT7* belongs to the α1,3/4-fucosyltransferase family, which includes FUT3, 4, 5, 6, 7, 9, 10, and 11, and catalyzes the last step in the synthesis of fucosylated glycoconjugates [17, 21]. Aberrant fucosylation has been implicated in tumor proliferation, metastasis and angiogenesis [22, 23]. Qin et al. reported that *FUT7* was expressed in follicular thyroid carcinoma (FTC) cells at higher level than in the paracancerous thyroid tissue, and it promoted the migration and invasion of FTC cells by activating MAPK and PI3K/Akt signaling pathways [20]. Liang et al. found that the expression of *FUT7* was elevated in A549 cells, and it played a vital role in cell growth and proliferation via triggering EGFR/AKT/mTOR signaling pathway [24]. In the study of Liu et al., *FUT7* promoted the proliferation, migration, invasion and epithelial–mesenchymal transition (EMT) of bladder cancer cells [25]. The methylation level of *FUT7* was decreased in bladder cancer tissues and reduced in patients with high stage status and nodal metastasis [25], indicating that *FUT7* methylation level might be a potential indicator reflecting clinical features of bladder urothelial carcinoma.

Epigenetic modifications are involved in the regulation of gene expression and the control of many cellular processes in both normal and cancer cells. UALCAN (http://ualcan.path.uab.edu/index.html), which could evaluate the potential role of DNA promoter methylation, showed that the methylation levels of *FUT7* in LUAD and LUSC were significantly lower than that in normal tissues (*P* < 0.05) (Additional file 1: Fig. S2). The dataset from Wanderer (http://maplab.imppc.org/wanderer/) demonstrated that the mean methylation level of each CpG site in the *FUT7* gene (chr9: 139927000–139928000) was significantly decreased in LUAD and LUSC compared to normal tissues (*P* < 0.05) (Additional file 1: Fig. S3). In general, our results were consistent with those of the database. We found seven CpG sites in chr9:139,927,462–139,927,771 by EpiTyper assay, and the results illustrated that the methylation levels of seven *FUT7* CpG sites in the blood of lung cancer were significantly lower than those in normal control (Table 2), and the hypomethylation of *FUT7* was associated with increased risk of lung cancer (Table 3). Moreover, the methylation levels of FUT7_CpG_2,3,5,6,7 decreased in terms of advanced stage (stage III and IV) and nodal metastasis of LC patients (Table 7). We speculated that various cancer types may share the same blood-based DNA methylation genes, but there are differences in methylation levels and specific sites due to changes in cancer types. Previous studies were performed mostly in the context of comparing cancers and healthy controls, while our study included benign pulmonary nodule. The hypomethylation levels of FUT7_CpG_4,5 were associated with increasing risk of LUAD, while the increased risk of LUSC was associated with the decreased methylation levels of FUT7_CpG_1,2,3,4,7 (Table 5). These results suggested that *FUT7* methylation could be used to differentiate lung cancer subtypes from BPN. Therefore, the specific molecular mechanisms between *FUT7* methylation and LC might be the subject for further research. The expression of *FUT7* in LC patients could provide additional hints for the regulation of DNA methylation. We suggested that the hypomethylation of *FUT7* may upregulate the function of *FUT7* and subsequently accelerate downstream pathways.

LUAD and LUSC are the largest non-small cell lung cancer (NSCLC) subgroups. In our present study, the levels of *FUT7* methylation were lower in LUSC patients than that in LUAD patients (Table 4; Fig. 3), and *FUT7* methylation indicated good diagnostic accuracy in the discrimination of LUSC versus NC or BPN (Fig. 2D, F). Experimental evidence suggested that LUAD and LUSC were vastly different in molecular, pathological and clinical levels. Genes differentially expressed between LUAD and LUSC contain main Gene Ontology subgroups [26–30], including the regulatory network of cell proliferation, DNA replication, DNA repair and RNA splicing. Different driver gene changes are related to different tumor diseases and distinct cell control pathways [31, 32]. In lung cancer, the types of mutated oncogenes and cells of origin decide the formation of LUAD versus LUSC, tumor invasiveness and aggressiveness. For instance, mutations in receptor tyrosine kinases are usual in LUAD but rare in LUSC [33]. The overexpression of keratins is connected with tumor progression in LUAD [34]. It is found that driving the p53/p63/p73 axis is closely associated with LUSC [35, 36], but not with LUAD. In histopathology, LUAD and LUSC have different origins. LUAD originates from cells secreting surfactant components, while LUSC originates from cells lining inside of the lung airways. LUAD is the most common type of lung cancer seen in nonsmokers and is more general in women than in men. Our results showed that LUAD patients were younger and more likely to be women compared with LUSC. LUAD has replaced LUSC as the most common histological subtype for unknown reasons in the past 25 years. LUSC is linked to a history of smoking and is frequently found in the main bronchus of the lungs. As the genetic drivers and tumor control networks are obviously different in LUAD and LUSC, further research works are needed to explain the specific mechanism for *FUT7* methylation in the progress of LUAD and LUSC.

In present cohort study, the sample size was large and diverse, covering normal control, benign pulmonary nodule and lung cancer patients. Moreover, multiple logistic regression was utilized to analyze the relationship between *FUT7* methylation level and lung cancer. There is no doubt that there are some limitations in our study. The sample size of each subtype of LC is small, and more LC patients of different subtypes are needed to determine the detection ability of *FUT7* methylation in them. Meanwhile, further functional studies are needed to explain the molecular mechanism for *FUT7* methylation in the progress of LC.

## Conclusions

In conclusion, this study disclosed a significant correlation between altered blood-based *FUT7* methylation and lung cancer, especially in LUSC. Our results provide novel evidence that changes DNA methylation in peripheral blood might be a potential biomarker for the evaluation of lung cancer risk.

## Supplementary Information

**Additional** **file** **1.** **Fig. S1**: The sequence of the *FUT7* amplicon. The *FUT7* amplicon examined by EpiTyper assay (chr9:139,927,462-139,927,771, antisense strand, build 37/hg19, in the UCSC Genome Browser). The measurable seven CpG sites by EpiTyper assay were in light gray. The numbers (50, 100,150, etc.) denoted number of bases per line. **Fig. S2**: Promoter methylation level of FUT7 in LUAD and LUSC in UALCAN. (A) Promoter methylation level of FUT7 in LUAD. (B) Promoter methylation level of FUT7 in LUSC.LUAD lung adenocarcinoma, LUSC lung squamous cell carcinoma. **Fig. S3**: Mean methylation of FUT7 in LUAD and LUSC in chr9: 139927000-139928000. (A) Mean methylation of FUT7 in LUAD. (B) Mean methylation of FUT7 in LUSC. LUAD lung adenocarcinoma, LUSC lung squamous cell carcinoma.

## Data Availability

The datasets used and/or analyzed during the current study are available from the corresponding author on reasonable request.
