# Supplementary material for: Novel blood-based FUT7 DNA methylation is associated with lung cancer: especially for lung squamous cell carcinoma
Source: Clin Epigenetics. 2022 Dec 3;14:167. doi: 10.1186/s13148-022-01389-2 (PMC9719144; doi:10.1186/s13148-022-01389-2)
Supplement: Supplementary file 1 — Additional file 1. Fig. S1: The sequence of the FUT7 amplicon. The FUT7 amplicon examined by EpiTyper assay (chr9:139,927,462-139,927,771, antisense strand, build 37/hg19, in the UCSC Genome Browser). The measurable seven CpG sites by EpiTyper assay were in light gray. The numbers (50, 100,150, etc.) denoted number of bases per line. Fig. S2: Promoter methylation level of FUT7 in LUAD and LUSC in UALCAN. (A) Promoter methylation level of FUT7 in LUAD. (B) Promoter methylation level of FUT7 in LUSC.LUAD lung adenocarcinoma, LUSC lung squamous cell carcinoma. Fig. S3: Mean methylation of FUT7 in LUAD and LUSC in chr9: 139927000-139928000. (A) Mean methylation of FUT7 in LUAD. (B) Mean methylation of FUT7 in LUSC. LUAD lung adenocarcinoma, LUSC lung squamous cell carcinoma. [file 13148_2022_1389_MOESM1_ESM.docx]

**Supplementary material**


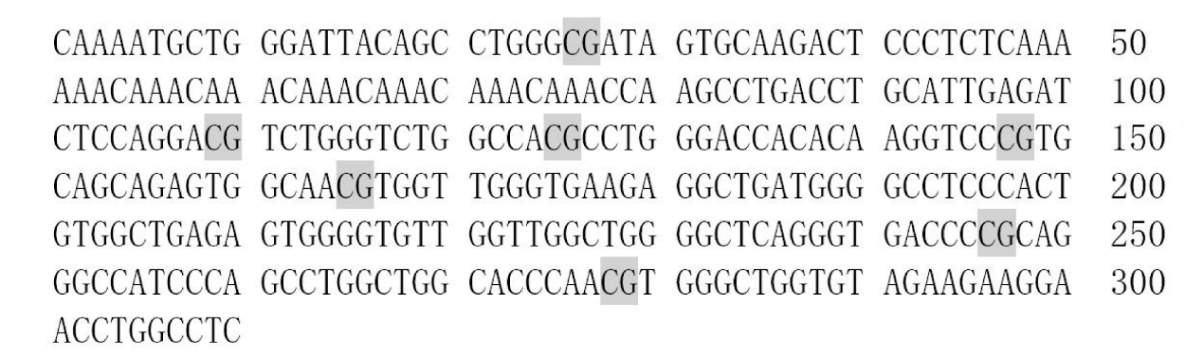


Figure S1 The sequence of the *FUT7* amplicon. The *FUT7* amplicon examined by EpiTyper assay (chr9:139,927,462-139,927,771, antisense strand, build 37/hg19, in the UCSC Genome Browser). The measurable seven CpG sites by EpiTyper assay were in light grey. The numbers (50, 100,150. etc.) denoted number of bases per line.

A


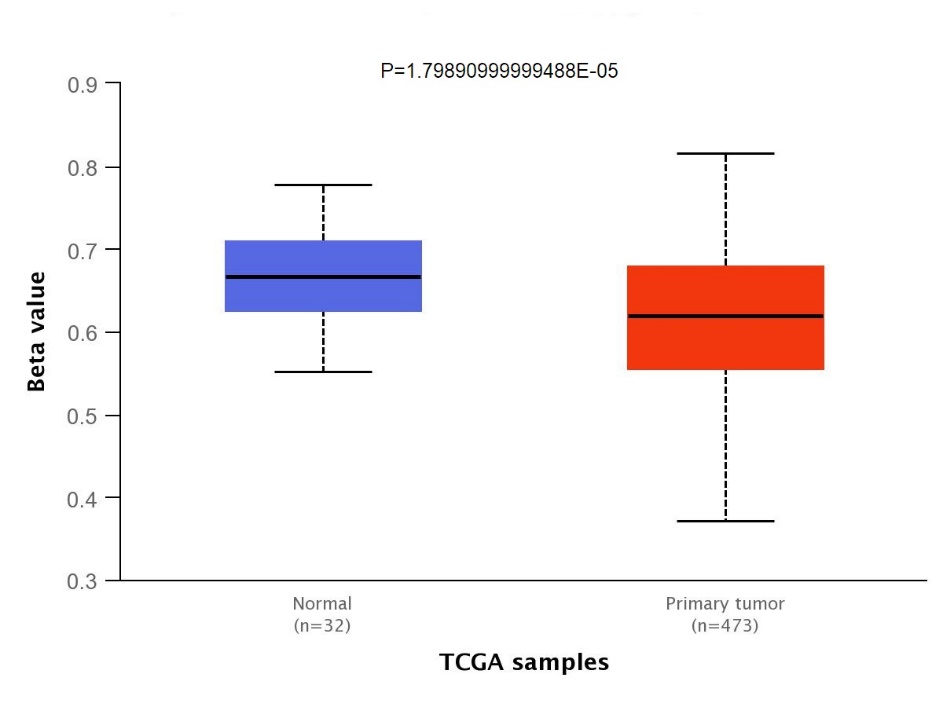


B


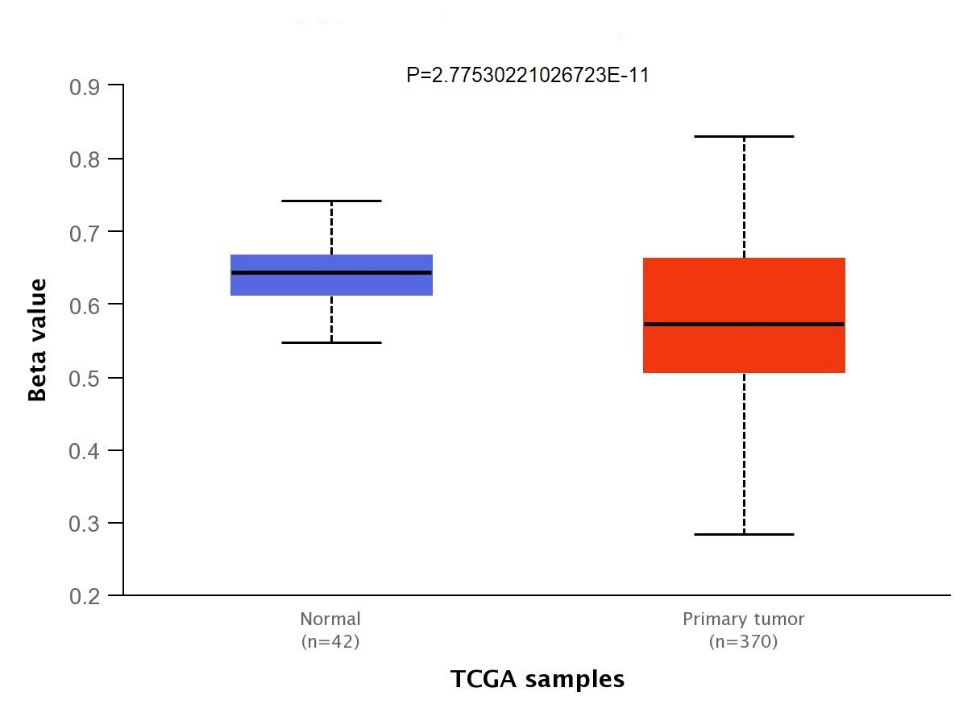


Figure S2 Promoter methylation level of FUT7 in LUAD and LUSC in UALCAN

(A) Promoter methylation level of FUT7 in LUAD. (B) Promoter methylation level of FUT7 in LUSC.LUAD lung adenocarcinoma, LUSC lung squamous cell carcinoma.

A


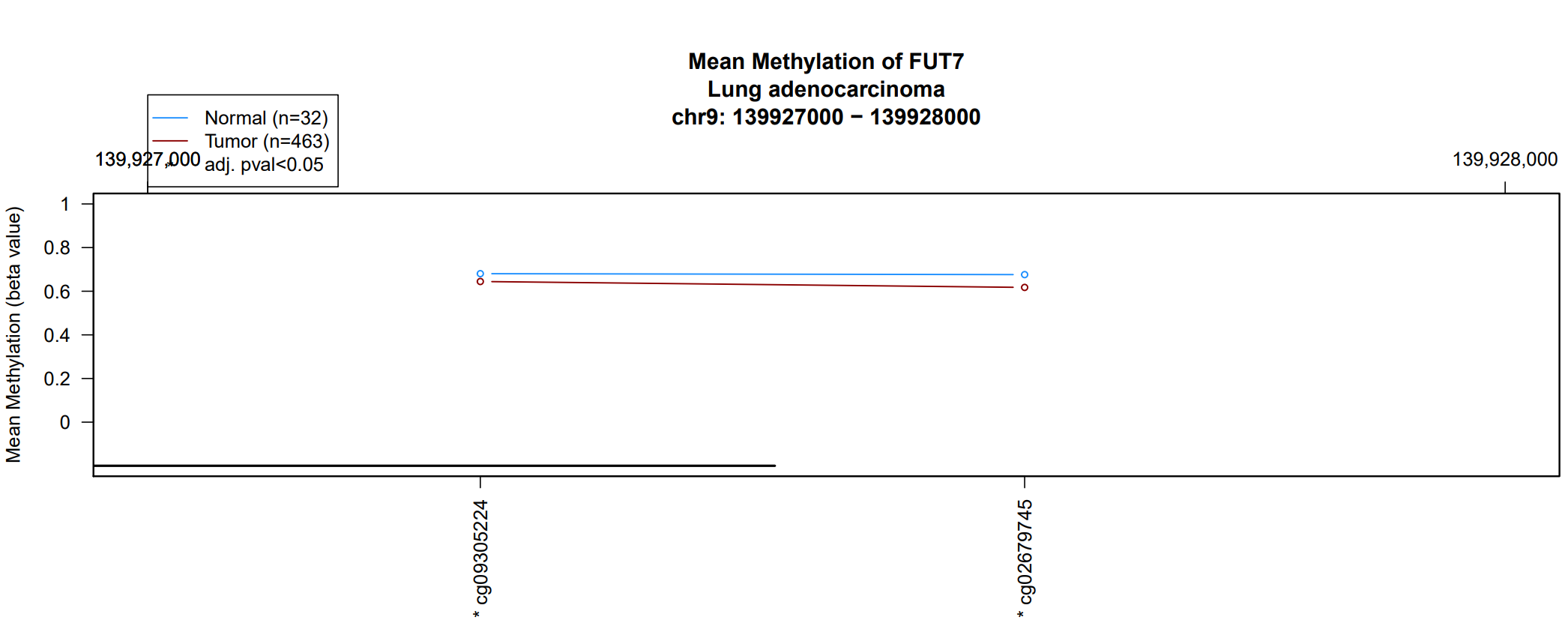


B


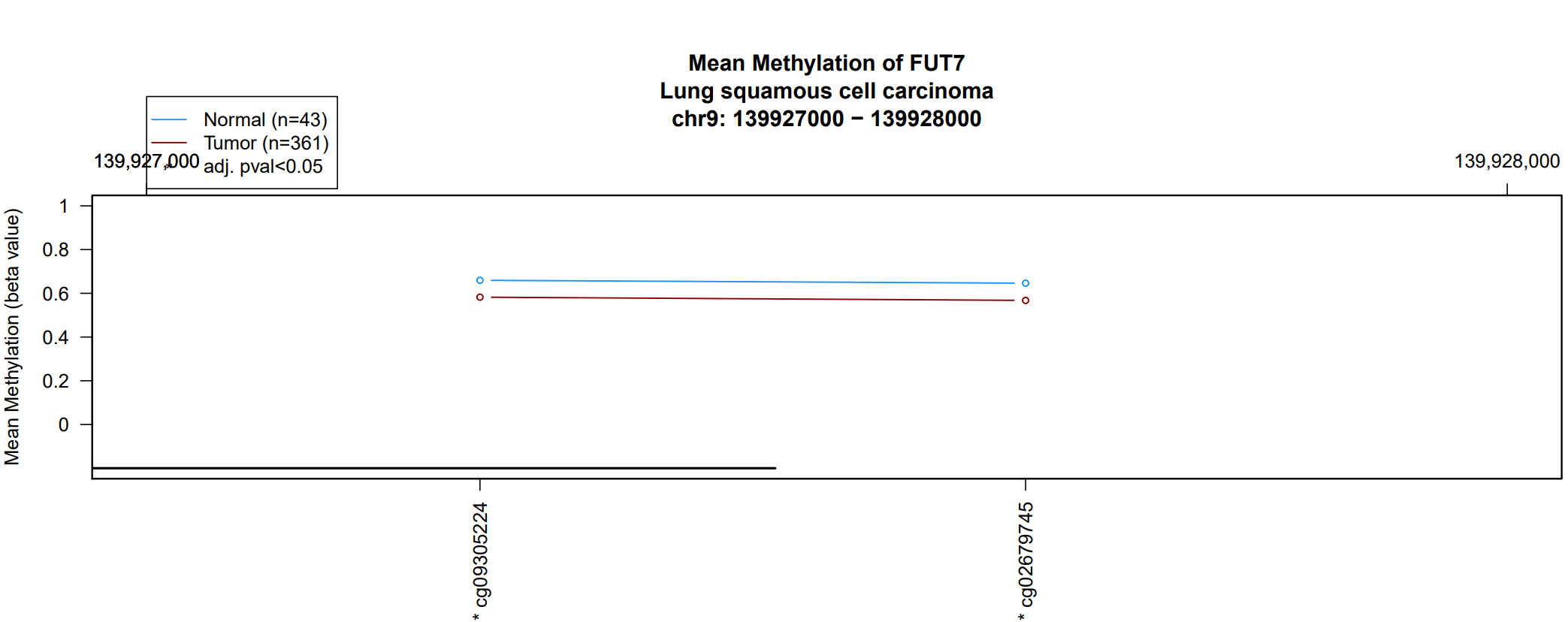


Figure S3 Mean methylation of FUT7 in LUAD and LUSC in chr9: 139927000-139928000

(A) Mean methylation of FUT7 in LUAD. (B) Mean methylation of FUT7 in LUSC. LUAD lung adenocarcinoma, LUSC lung squamous cell carcinoma.
